# A Machine Learning Approach for an Improved Inertial Navigation System Solution

**DOI:** 10.3390/s22041687

**Published:** 2022-02-21

**Authors:** Ahmed E. Mahdi, Ahmed Azouz, Ahmed E. Abdalla, Ashraf Abosekeen

**Affiliations:** Electrical Engineering Branch, Military Technical College, Kobry El-Kobba, Cairo 11766, Egypt; ahmedelgibly@yahoo.com (A.E.M.); a.azouz@mtc.edu.eg (A.A.); elsaid_a@mtc.edu.eg (A.E.A.)

**Keywords:** INS, MEMS-IMU, machine learning, ANFIS, positioning, navigation

## Abstract

The inertial navigation system (INS) is a basic component to obtain a continuous navigation solution in various applications. The INS suffers from a growing error over time. In particular, its navigation solution depends mainly on the quality and grade of the inertial measurement unit (IMU), which provides the INS with both accelerations and angular rates. However, low-cost small micro-electro-mechanical systems (MEMSs) suffer from huge error sources such as bias, the scale factor, scale factor instability, and highly non-linear noise. Therefore, MEMS-IMU measurements lead to drifts in the solutions when used as a control input to the INS. Accordingly, several approaches have been introduced to model and mitigate the errors associated with the IMU. In this paper, a machine-learning-based adaptive neuro-fuzzy inference system (ML-based-ANFIS) is proposed to leverage the performance of low-grade IMUs in two phases. The first phase was training 50% of the low-grade IMU measurements with a high-end IMU to generate a suitable error model. The second phase involved testing the developed model on the remaining low-grade IMU measurements. A real road trajectory was used to evaluate the performance of the proposed algorithm. The results showed the effectiveness of utilizing the proposed ML-ANFIS algorithm to remove the errors and improve the INS solution compared to the traditional one. An improvement of 70% in the 2D positioning and of 92% in the 2D velocity of the INS solution were attained when the proposed algorithm was applied compared to the traditional INS solution.

## 1. Introduction

With the advantages of being a self-contained system and providing an uninterrupted navigation solution, the inertial navigation system (INS) has become an essential component to obtain a robust navigation solution in several fields such as aircraft applications, autonomous navigation, and vehicle dynamic control [1]. Despite the advantage of the INS having a high short-term accuracy, it suffers from the drift accumulation of the biases over time. The accuracy of the INS’s navigation solution and the ability to reduce the errors accumulated over time depend on the type of inertial measurement unit (IMU) [2,3]. Recently, the utilization of micro-electro-mechanical systems (MEMSs) has been introduced for inertial sensor systems with the advantages of low cost, small size, and low power consumption [4]. On the other hand, the disadvantage of the high error accumulation rate of MEMSs has raised the challenge of modeling these errors to improve the accuracy of the navigation solution [5].

The difficulty of modeling these errors is due to the existence of non-linear errors. These errors cannot be modeled by the traditional techniques such as the Kalman filter (KF), the extended KF (EKF), the unscented Kalman filter (UKF), or even the particle filter (PF) [3,6,7]. Accordingly, there is a great need to find an alternative to traditional methods that does not have the difficulty and complexity of error modeling. Therefore, researchers have taken advantage of the availability of a large amount of data extracted from the INS and added machine learning (ML) techniques to the navigation algorithms [6,8,9,10].

ML techniques are utilized as estimators/predictors or classifiers of the navigation parameters. These techniques are utilized to smooth the choice of the sensors as an alternative to the KF in a plug-and-play manner [6,8]. This leads to the selection of the integration process and the raw measurements. Consequently, training the ML model helps produce a robust predictive model for the INS errors during GNSS outages. In addition, it can be used to improve visual positioning, mitigating the non-line-of-sight (NLOS) effects such as the multipath effect, spoofing, and jamming [11].

There is growing interest in utilizing ML techniques to improve the INS navigation solution. An approach utilizing an artificial neural network (ANN) to overcome the limitations of the KF to bridge the GPS outages during the GPS/INS integration process was introduced in [12,13,14,15]. The proposed methodology was accomplished in two phases. In the first phase, the ANN was trained to predict the INS position error and remove it from the corresponding INS position without having the initial position of the INS. Furthermore, the work in [16,17] utilized the ANN and ANFIS after the GPS/INS integration to enhance the INS navigation solution. In contrast, the work in [18] introduced a non-linear autoregressive neural network with external inputs (NARX) combined with the UKF to enhance the position and velocity accuracy of the INS/GNSS integration. Furthermore, the work in [3] proposed a fast orthogonal search (FOS) model to reduce and compensate the unmodeled residual non-linear errors of a mag/radar/RISS/GPS integration system to improve the navigation solution during GPS outages. The work in [19] utilized the FOS model as a GPS swept anti-jamming technique to discriminate between the authentic GPS signal and the interference from the chirp frequency jammer. In [20], two approaches were introduced to overcome the drift during GNSS outages using parallel cascaded mechanization for non-linear error estimation of the INS solution. The results showed a slight improvement during the parts of the trajectory that had maneuvers such as turns, while the parts with few maneuvers had a significant improvement. The work in [21] proposed a random forest (RF) method for standstill recognition. The proposed method depends on the generated features from the IMU signals that represent the standstill state as an input for the classifier.

In comparison, the work in [22] introduced a supervised machine learning technique for spoof detection. The work in [23] introduced an adaptive fuzzy extended Kalman filter (AFEKF) to enhance the prediction level of the position and velocity errors of the INS. In [6,8], the authors introduced a sensor fusion technique based on fuzzy clustering to fuse the Doppler speed from an FMCW radar and the speedometer data to improve the input speed of an RISS model. The results showed the enhancement of the navigation solution in some portions of the trajectory. On the other hand, the lack of sensor fault detection and a false reading algorithm caused a drift in some portions that had wheel slippage. The work in [24] proposed a fuzzy cluster means (FCMs) technique to fuse multiple IMUs to produce a robust measurement, which was utilized in INS mechanization integrated with GPS. The results in this work showed significant improvement when using FCMs with a multi-IMU structure compared to using only one. Furthermore, the work in [25] utilized the ANFIS model to predict the dual-mass MEMS gyroscope’s output drift caused by temperature. The work in [26] utilized the ANFIS model to enhance the navigation solution of the INS by training the ANFIS model on a differential GPS dataset as a reference position and evaluated the model on a raw public dataset (KITTI) with a trajectory that lasted from (140–300) s. Furthermore, the work in [27] utilized the ANFIS model as a solution for the navigation problem of a mobile robot.

The work in [28] utilized empirical mode decomposition threshold filtering (EMDTF) and a long short-term memory (LSTM) neural network. The EMDTF disposes of the noise generated in the INS’s sensors, while the LSTM is used to predict the pseudo-GPS position during GPS outages. The presented EMDTF scheme improved the accuracy of east velocity, north velocity, longitude, and latitude by 9.12%, 15.14%, 13.78%, and 10.72%, respectively, while the LSTM scheme reduced the RMSE by 21.79%, 14.85%, 55.03%, and 19.66% over the traditional artificial neural networks. Moreover, the work in [29] overcame the dilemma of poor navigation accuracy in challenging environments by proposing a fusion scheme utilizing machine learning techniques. The proposed scheme utilizes the support vector regression-based adaptive Kalman filter (SVR-AKF) to regulate the covariance parameters of the KF. In addition, the adaptive neuro-fuzzy inference system (ANFIS) was used to predict the navigation solution errors of the INS during GNSS outages. The proposed scheme was compared to the traditional schemes using the KF and EKF over two real trajectories. The results showed an improvement in the position error of about 58.8% against the KF over Trajectory 1and 48% to 67.5% against the KF and 34.2% to 57.6% against the EKF over Trajectory 2. Another approach using the FIS to adapt the fuzzy covariance matrix for the online calibration of multiple LiDAR systems was presented in [30]. The aim of this work was enhancing the performance of low-cost laser sensors, and a minimum error for distance of 2.8 cm and a rotation of 1.2 degrees were obtained.

A recent survey of ML techniques and how they can be involved in all the fundamental steps of inertial sensing applications to improve the navigation solution obtained from the INS was provided in [31], stating the advantages and the challenges. The authors mentioned several challenges that the use of ML faces with regard to inertial sensors such as the nonexistence of hardware combinations of inertial sensors and ML and the lack of work on the sensor measurements’ improvement using ML. In addition, the authors mentioned that the use of ML along with the sensor measurements is a promising field of research, as most of the work conducted on ML has only been on the INS navigation solution.

From the discussion of the previous related work, we noticed that most of the presented research utilized ML techniques for the INS solution, but neglected the inertial sensors’ measurements. Therefore, in this paper, we propose an ANFIS algorithm to be applied to the raw measurements of a commercial IMU to leverage its performance. This process was carried out using a high-end IMU as a reference to provide a suitable model for the low-end IMU in the ML structure. The model was generated in the training phase using both IMUs, then applied only to the low-end IMU in the testing phase. The proposed ML algorithm was evaluated on a real road trajectory. The results showed a significant improvement of the commercial IMU measurements, as well as the INS navigation solution compared to the traditional INS solution. The contributions of this research paper are summarized as follows:

1. The development of an ML-based ANFIS algorithm as an ML technique to leverage a low-grade IMU;
2. Comparing the low-grade IMU measurements before and after applying the proposed algorithm to the reference IMU;
3. The validation of the proposed algorithm by applying the tested IMU data to the INS mechanization.

This paper is organized into six sections. In Section 1, the introduction, related work, and paper contributions are discussed. Section 2 gives the background of the INS and the ANFIS algorithm. The methodology is explained in Section 3. The experimental setup and the utilized units are detailed in Section 4. The results are discussed in Section 5. Finally, the paper is concluded in Section 6.

## 2. Background

### 2.1. Inertial Navigation Systems

**Figure 1:**
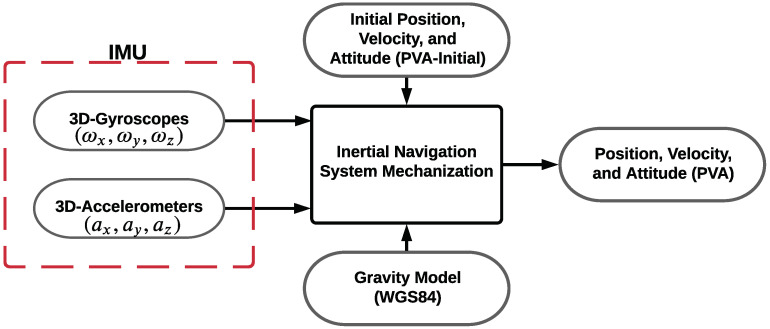
Strap down INS block diagram.

The traditional inertial navigation system is composed of an IMU and a navigation processor. The IMU is composed of three accelerometers providing the specific forces and three gyroscopes providing the angular rates [1,32,33,34,35,36], as shown in Figure 1.

The INS depends on the knowledge of the target’s initial states (position, velocity, and attitude (PVA)) and updates its current states accordingly, as shown in Equations (1)–(3).

The mechanization process of the INS can be summarized in three main steps. First, we obtained the angular rates ($\omega_{x},\omega_{y},\omega_{z}$) from the gyroscopes, the accelerations ($f_{x},f_{y},f_{z}$) from the accelerometers, and the attitude angles of the pitch, roll, and yaw (p,r,y) from the angular rates after calculating the transformation matrix. Second, with the assistance of the rotation matrix, the forces in the navigation frame from the body frame can be obtained and then transformed to the local-level frame (LLF). Finally, the velocity was obtained by integrating the transformed forces, and the position was obtained by integrating the calculated velocity [1,37].

(1) $$P={\left[\varphi \;\;\lambda \;\;h\right]}^{T}$$

where *P* is the position, φ is the latitude, λ is the longitude, and h is the altitude.

(2) $$V={\left[V_{N}{\;V}_{E}\;V_{D}\right]}^{T}$$

where *V* is the velocity, $V_{N}$ is the north velocity, $V_{E}$ is the east velocity, and $V_{D}$ is the down velocity.

The attitude is determined by Equation (3) [35].

(3) $$A=\left[\begin{array}{c} q_{0}\\ q_{1}\\ q_{2}\\ q_{3} \end{array}\right]$$

where *A* is the quaternion representation of the attitude and the quaternions $q_{0},q_{1},q_{2},$ and $q_{3}$ are the parameters of the rotation matrix.

The attitude rates are calculated as Equation (4).

(4) $$\left[\begin{array}{c} \dot{q_{0}}\\ \dot{q_{1}}\\ \dot{q_{2}}\\ \dot{q_{3}} \end{array}\right]=0.5\left[\begin{array}{cccc} 0 & \omega_{x} & -\omega_{y} & -\omega_{z}\\ \omega_{x} & 0 & \omega_{z} & -\omega_{y}\\ \omega_{y} & -\omega_{z} & 0 & \omega_{x}\\ \omega_{z} & \omega_{y} & -\omega_{x} & 0 \end{array}\right]\left[\begin{array}{c} q_{0}\\ q_{1}\\ q_{2}\\ q_{3} \end{array}\right]$$

where $\omega_{x}$, $\omega_{y}$, and $\omega_{z}$ are the gyroscopes’ angular rates in the *x*, *y*, and *z* directions, respectively.

Furthermore, the quaternion attitude can be transferred to the Euler angles of the roll, pitch, and yaw, respectively, as in Equation (5).

(5) $$\left[\begin{array}{c} \phi \\ \theta \\ \psi \end{array}\right]=\left[\begin{array}{c} \left.atan2\left(2\left.\left.q_{2}q_{3}\right.+\left.{2q}_{1}q_{0}\right.\right.),\left(q_{3}^{2}\right.+\left.q_{0}^{2}\right.-\left.q_{1}^{2}\right.-\left.q_{2}^{2}\right)\right)\right.\\ -asin\left(2\left.\left.q_{1}q_{3}\right.-\left.{2q}_{2}q_{0}\right.\right.\right)\\ atan2\left(\left(2\left.\left.q_{1}q_{2}\right.+\left.{2q}_{0}q_{3}\right.\right.\right),\left(q_{0}^{2}\right.+\left.q_{1}^{2}\right.-\left.q_{2}^{2}\right.-\left.q_{3}^{2}\right)\right) \end{array}\right]$$

where ϕ is the roll angle in radians, θ is the pitch angle in radians, ψ is the yaw angle in radians.

Equation (6) shows the transformation matrix from the body frame to the LLF using quaternion states [38].

(6) $$C_{b}^{n}=\left[\begin{array}{ccc} q_{1}^{2}+q_{0}^{2}-q_{2}^{2}-q_{3}^{2} & 2(q_{1}q_{2}-q_{3}q_{0}) & 2(q_{2}q_{3}+q_{2}q_{0})\\ 2(q_{1}q_{2}+q_{3}q_{0}) & q_{2}^{2}+q_{0}^{2}-q_{1}^{2}-q_{3}^{2} & 2(q_{2}q_{3}-q_{1}q_{0})\\ 2(q_{1}q_{3}-q_{2}q_{0}) & 2(q_{2}q_{3}+q_{1}q_{0}) & q_{3}^{2}+q_{0}^{2}-q_{1}^{2}-q_{2}^{2} \end{array}\right]$$

The specific forces can be transformed into the LLF using the transformation matrix $C_{b}^{n}$ and are obtained with Equation (7).

(7) $$\left[\begin{array}{c} F_{N}\\ F_{E}\\ F_{D} \end{array}\right]=\;C_{b}^{n}\left[\begin{array}{c} f_{x}\\ f_{y}\\ f_{z} \end{array}\right]$$

where $F_{N}$, $F_{E}$, and $F_{D}$ are the transformed specific forces in the north, east, and down frame, respectively.

The velocity rates can be obtained with Equation (8).

(8) $$\begin{array}{c} \left[\begin{array}{c} {\dot{V}}_{N}\\ {\dot{V}}_{E}\\ {\dot{V}}_{D} \end{array}\right]=\left[\begin{array}{ccccccc} 1 & 0 & 0 & 0 & -\left(\dot{\lambda }+2w_{e}sin\left(\varphi \right)\right) & \dot{\varphi } & 0\\ 0 & 1 & 0 & \left(\dot{\lambda }+2w_{e}sin\left(\varphi \right)\right) & 0 & \left(\dot{\lambda }+2w_{e}cos\left(\varphi \right)\right) & 0\\ 0 & 0 & 1 & -\dot{\varphi } & -\left(\dot{\lambda }+2w_{e}cos\left(\varphi \right)\right) & 0 & 1 \end{array}\right]\left[\begin{array}{c} F_{N}\\ F_{E}\\ F_{D}\\ V_{N}\\ V_{E}\\ V_{D}\\ g \end{array}\right] \end{array}$$

where $\dot{\lambda }$ and $\dot{\varphi }$ are the longitude and latitude rates, respectively, $w_{e}=7.2921158\times 10^{-5}$ rad/s is the magnitude of the rotation rate of the Earth, and *g* is the acceleration due to gravity, which can be obtained with Equation (9).

(9) $$g=\;g_{WGS0}\frac{1+g_{WGS1}sin\left(\varphi \right)}{{\left[1-\;E^{2}{sin}^{2}\left(\varphi \right)\right]}^{\frac{1}{2}}}-\left[3.0877\times 10^{-6}-0.0044\times 10^{-6}{sin}^{2}\left(\varphi \right)\right]h+0.072\times 10^{-12}$$

where $g_{WGS0}=9.78032677$ m/s${}^{2}$ is the gravity at the Equator, $g_{WGS1}=0.00193185138639$ m/s${}^{2}$ is the gravity formula constant, and E=0.0818191908426 is the first eccentricity [1,2].

The position rates are obtained with Equation (10).

(10) $$\left[\begin{array}{c} \dot{\varphi }\\ \dot{\lambda }\\ \dot{h} \end{array}\right]\;=\;\left[\begin{array}{c} \frac{V_{N}}{R_{M}+h}\\ \frac{V_{E}}{\left(R_{N}+h\right)cos\left(\varphi \right)}\\ {-V}_{D} \end{array}\right]$$

where $R_{M}$ and $R_{N}$ are the meridian radius and normal radius of the Earth’s ellipsoid model, respectively.

Unfortunately, the INS suffers from error growth over time because of the two-times integration process of the target’s acceleration. The errors in the INS can be categorized and divided into deterministic and stochastic errors. The deterministic errors include the bias offset, scale factor, and axis misalignment errors. In contrast, the stochastic errors include bias drift, bias stability, scale factor stability, noise, and axis misalignment errors. The deterministic errors can be reduced or compensated if the sensors are properly calibrated, especially high-end sensors, while the stochastic errors were modeled randomly to reduce their effect [30]. Therefore, the gyroscope measurement model is represented by Equation (11).

(11) $${\tilde{\omega }}_{ib}^{b}=\omega_{ib}^{b}+\;b_{g}+\;S\omega_{ib}^{b}+N\omega_{ib}^{b}+\varepsilon_{g}$$

where ${\tilde{\omega }}_{ib}^{b}$ is the gyroscope measurement vector, $\omega_{ib}^{b}$ is the true angular rate velocity vector, $b_{g}$ is the gyroscope instrument bias vector, $S_{g}$ is a matrix representing the gyro scale factor, $N_{g}$ is a matrix representing the non-orthogonality of the gyro triad, and $\varepsilon_{g}$ is the vector representing the gyro sensor noise.

Furthermore, the accelerometers’ measurement model is represented by Equation (12).

(12) $${\tilde{f}}^{b}=f^{b}+\;b_{a}+\;S_{1}f+S_{2}f^{2}+\;N_{a}f+\delta_{g}+\eta_{g}$$

where ${\tilde{f}}^{b}$ is the accelerometer measurement vector, $f^{b}$ is the true specific force vector, $b_{a}$ is the accelerometer instrument bias vector, $S_{1}$ is a matrix of the linear scale factor error, $S_{2}$ is a matrix of the non-linear scale factor error, $N_{a}$ is a matrix representing the non-orthogonality of the accelerometer’s triad, $\delta_{g}$ is the anomalous gravity vector, and $\eta_{g}$ is a vector representing the accelerometer sensor noise.

The classification of the INS depends on the IMU’s accuracy and its ability to reduce the error growth over time. Therefore, to compensate the low-cost commercial IMU’s errors, either traditional techniques or ML techniques are applied to enhance the INS’s navigation solution.

### 2.2. Adaptive Neuro-Fuzzy Inference System

The adaptive neuro-fuzzy inference system (ANFIS) is a fusion technique between the artificial neural network (ANN) and the fuzzy inference system (FIS). Subsequently, it provides the advantages of both techniques and compensates their disadvantages. It drives the system to adapt through the self-organizing and self-learning process [39].

**Figure 2:**
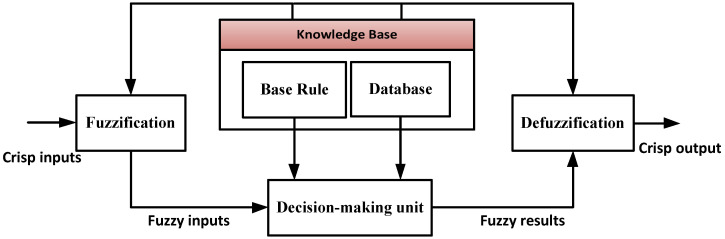
The structure of the fuzzy inference system.

The main structure of the FIS is shown in Figure 2. The FIS is based on the fuzzy conditional statements (if–then rules), which are responsible for making the decisions in an uncertain environment and its influencing factors [40]. The structure of the FIS is composed of five main blocks. The fuzzification process switches the crisp inputs into datasets by applying the membership function (MF). The base rule contains the fuzzy if–then rules, and the database comprises the MF utilized in the fuzzy rules. The decision-making unit executes the inference operation on the fuzzy rules. The defuzzification process turns the fuzzy results into a crisp output [41,42,43].

**Figure 3:**
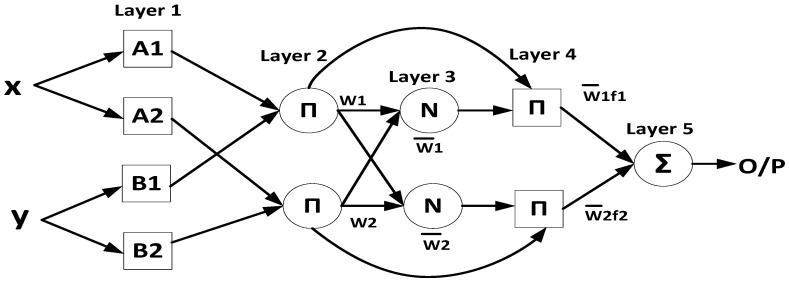
The ANFIS’s structure [44].

Similarly, ANFIS’s functionality is equivalent to the FIS, as shown in Figure 3 [44]. The ANFIS structure is composed of five layers. In the first layer, each node assigns the crisp inputs after applying the MF. The output of this layer clarifies how well the input matchesthe linguistic label, as given by Equation (13) [40,42].

(13) $$O_{i}^{1}=\mu_{A_{i}}\left(x\right)$$

where $\mu_{A_{i}}$ is the MF, *X* is the input to node *i*, and $A_{i}$ is the linguistic label for input *X*.

The second layer multiplies the input signals at each node to obtain the rules’ weights (firing strength), as given in Equation (14).

(14) $$W_{i}=\mu_{A_{i}}\left(x\right)\times \mu_{B_{i}}\left(y\right)$$

where x,y are the two inputs, $A_{i}$ is the linguistic label for input *X*, and $B_{i}$ is the linguistic label for input *y*. The third layer normalizes the weights of each rule by computing the ratio of the weight of each rule to the sum of all the rules’ weights, as given in Equation (15).

(15) $$\tilde{W}\;=\;\frac{W_{i}}{\sum W}$$

In the fourth layer, the normalized weights of each rule are multiplied by the output of the second layer, as given in Equation (16).

(16) $$O_{i}^{4}\;=\;\tilde{W_{i}}f_{i}\;=\;\tilde{W_{i}}\left(p_{i}x+q_{i}y+r_{i}\right)\;$$

where $\tilde{W_{i}}$ is the normalized weight obtained from the third layer and $p_{i},q_{i},$ and $r_{i}$ are called the consequent parameters.

Finally, the fifth layer sums all the incoming signals to compute the overall output $O_{f}$, as given in Equation (17).

(17) $$O_{f}\;=\;\sum_{i}\tilde{W}f_{i}\;=\;\frac{\sum_{i}W_{i}f_{i}}{\sum_{i}W_{i}}$$

## 3. Methodology

As mentioned in the previous section, the accuracy of the INS’s navigation solution depends on the quality/grade of the IMU sensor and the ability to compensate its errors. In this paper, we exploited the capability of the ML-ANFIS technique to estimate the inertial sensors’ errors by training a low-grade IMU with a high-end one. This work aimed to boost the low-grade IMU’s performance.

**Figure 4:**
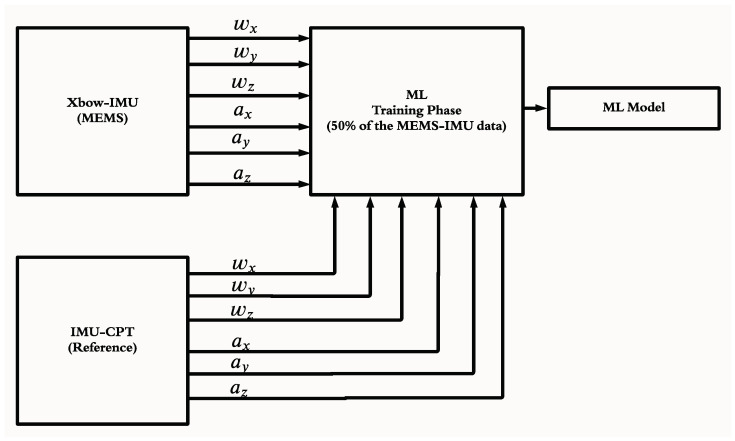
The block diagram of the training phase of the ML-based-ANFIS showing the model generation process.

The proposed ML technique is composed of two phases, the training phase and the testing phase. The training phase block diagram is shown in Figure 4. In this phase, the training dataset consisted of the low-grade IMU’s sensor measurements as the input and the high-grade IMU as the output. This phase was carried out using half of the trajectory data. The triangular and Gaussian MFs were utilized. In this paper, six triangular MFs were utilized as the ANFIS input layer for each IMU measurement. The triangular MF is simpler and faster to implement compared to other MFs such as the Gaussian MF [45,46]. Subsequently, the rule base contains one rule for every input MF combination. The clustering method utilized was the “grid partition”, in which every input variable is equally distributed over the input MF and generates a single-output “Sugeno fuzzy system”. The output ANFIS layer utilizes linear MFs in which the output of every rule is linearly related to the input variables and scaled by the previous result’s value. Finally, a thousand iterations were used to produce the model, which was applied later to the IMU measurements for the testing phase.

**Figure 5:**
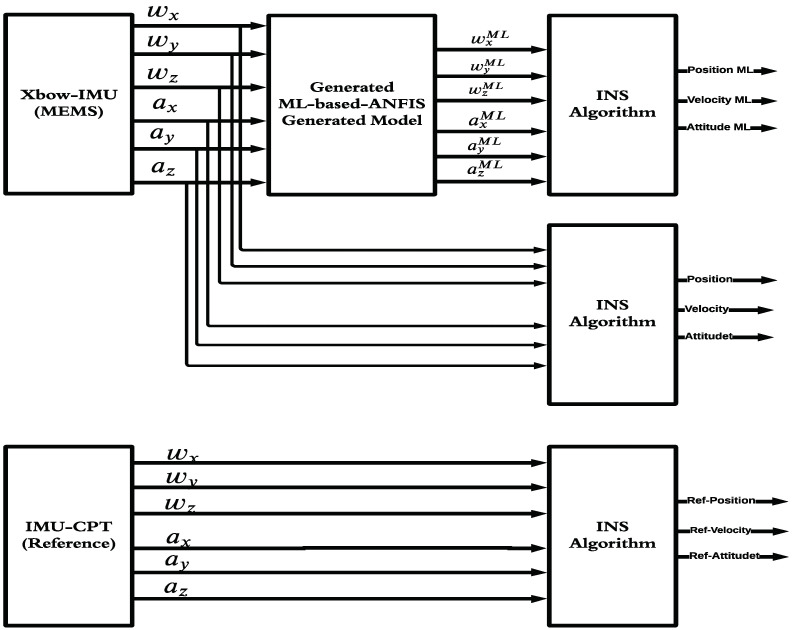
The block diagram of the testing phase of the ML-based-ANFIS showing the application of the generated model to the XBOW-IMU and comparing the produced PVA with the reference IMU.

The testing phase is shown in Figure 5. The ML model generates its predicted IMU measurements (six sensor measurements) to obtain the position, velocity, and attitude (PVA). Then, the navigation solution of the ML model is compared to the navigation solution of the reference to obtain the ${\Delta PVA}_{ML}$ of the ML model. Similarly, the navigation solution of the low-grade IMU is compared to the navigation solution of the reference to obtain the ΔPVA of the low-grade IMU. The differences in the errors in the navigation solution between the ML model and the low-grade IMU were calculated to compute the influence of the ML model in enhancing the navigation solution of the low-grade IMU’s sensor measurements, which is shown in the upcoming sections.

The overall algorithm is explained in Algorithm 1 in pseudo-code form.

**Algorithm 1** INS Solution Improvement Using ML
**Input**
IMU’s sensor measurements of three gyroscopes and three accelerometers $\left(\omega_{x},\;\omega_{y},\;\omega_{z},\;a_{x},\;a_{x},\;a_{x}\right)$ for the MEMS-IMU and the reference IMU, initial PVA states $\left({Lat}_{0},{\;Long}_{0},{Att}_{0},V_{0}^{N},V_{0}^{E},V_{0}^{D},\;p_{0},r_{0},y_{0}\right)$, and the navigation solution of the reference IMU (pos_ref,vel_ref,att_ref).
**Step 1**
Prepare and tune the ML-ANFIS options (input data, output data, type of clustering, MF type, number of Ms, F and epochs/iterations).
**Step 2**
Apply the ML-ANFIS on 50% of the input data (training phase).
**Step 3**
Generate the ML-ANFIS.
**Step 4**
Evaluate and apply the ML-ANFIS on the remaining data (testing phase).
**Step 5**
Evaluate the ML-ANFIS’s output (improved IMU sensor measurements $\left(\omega_{x},\omega_{y},\omega_{z},a_{x},a_{y},a_{z}\right)$.
**Step 6**
Compare the MEMS IMU’s sensor measurements and the ML-ANFIS IMU’s sensor measurements to the reference IMU’s sensor measurements to compute the percentage of improvement caused by the ML-ANFIS (RMSE). $RMSE=\sqrt{\frac{1}{n}\sum_{n}{\left(X_{n,Ref}-X_{n,ML}\right)}^{2}}$ where $X_{n,Ref}$ and $X_{n,ML}$ are the reference IMU and trained IMU measurements, respectively.
**Step 7**
Compute the ML-ANFIS’s navigation solution (PVA) by using the output of the ML-ANFIS as the input to the INS.
**Step 8**
Compare the MEMS IMU (PVA) and the ML-ANFIS (PVA) to the reference IMU (PVA) to compute the percentage of improvement of the ML-ANFIS (PVA) using the RMSE metric.
**Output**
The INS solution (PVA) of the MEMS-IMU and the ML model compared to the output using the reference IMU.

## 4. Experimental Setup

**Figure 6:**
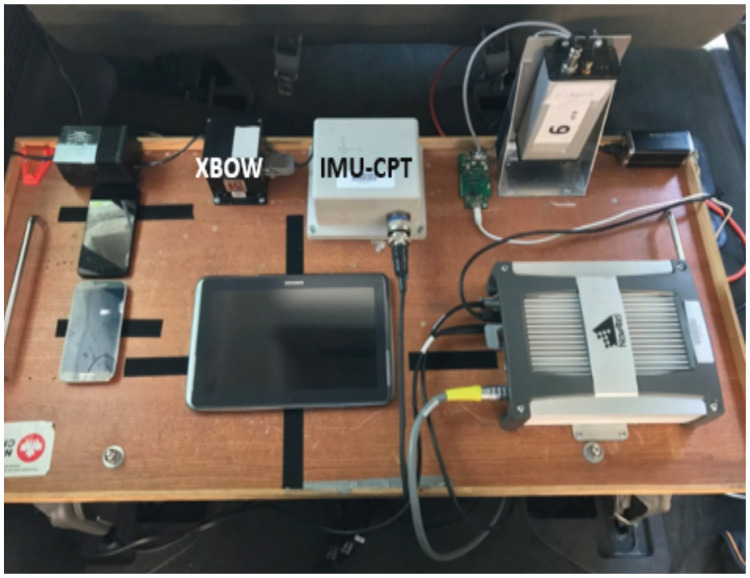
The utilized IMUs mounted on the testbed showing their placement and orientation inside the van.

The experimental work was carried out to verify the effectiveness of the ML model through a real road test trajectory with no pre-processing steps. The IMUs utilized in the experimental work were set up inside the test van as shown in Figure 6. The testbed was installed inside the van, coinciding with its axes. Furthermore, utilizing a standard seat chassis, the testbed was rigidly and firmly settled in the rear seat location. The low-grade IMU sensor utilized in this research was the Crossbow MEMS-grade XBOW IMU300CC, and the high-grade IMU utilized as a reference was the IMU-CPT, which includes three MEMS accelerometers and three fiber-optic gyroscopes (FOGs). The specifications of the two IMU units can be found in Table 1.

## 5. Results and Discussion

A real road trajectory was used to test the proposed ML technique’s performance in the downtown area of Kingston, ON, Canada. The reference trajectory was the INS solution that utilized the IMU-CPT sensor measurements as a control input to the IMS mechanization. Moreover, the trajectory lasted for 2300 s (almost 44 min) and contained various maneuvers at different speeds.

**Figure 7:**
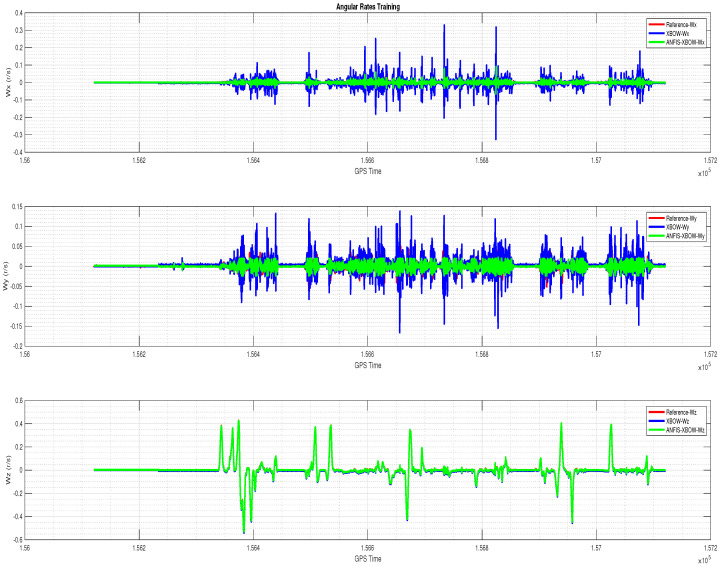
The 3D gyroscope angular rates with the ML–based–ANFIS (training stage).

**Figure 8:**
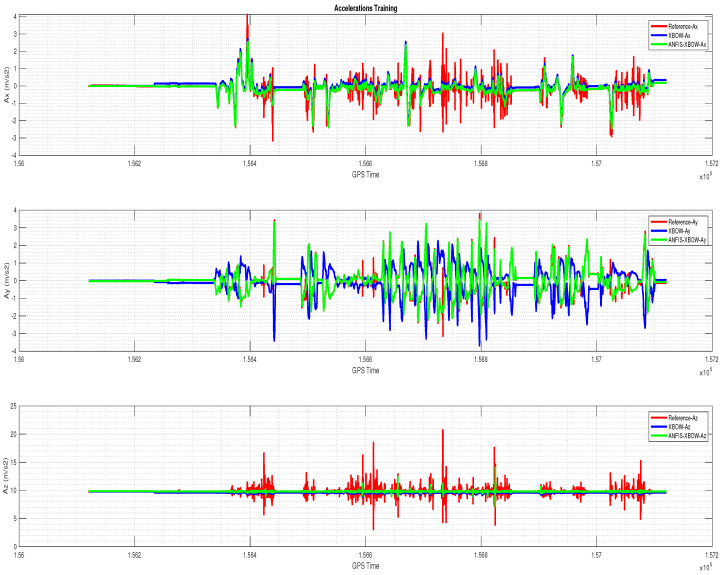
The 3D accelerometers with the ML–based–ANFIS (training stage).

**Figure 9:**
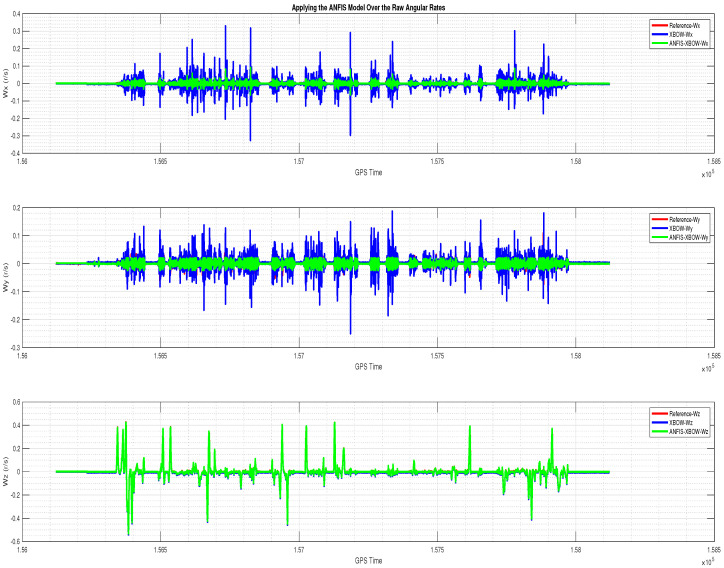
The 3D gyroscope angular rates after applying the ML–based–ANFIS (testing stage).

**Figure 10:**
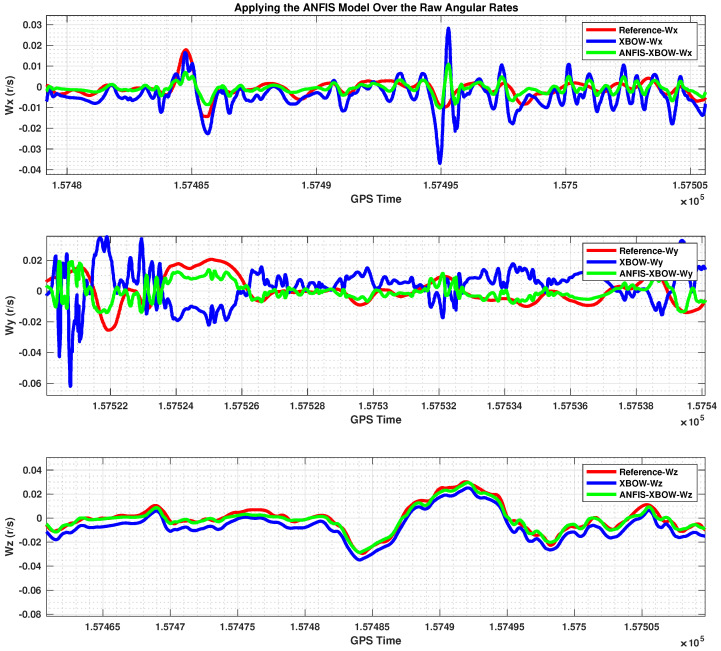
A zoomed–in part of the IMU gyroscope measurements.

**Figure 11:**
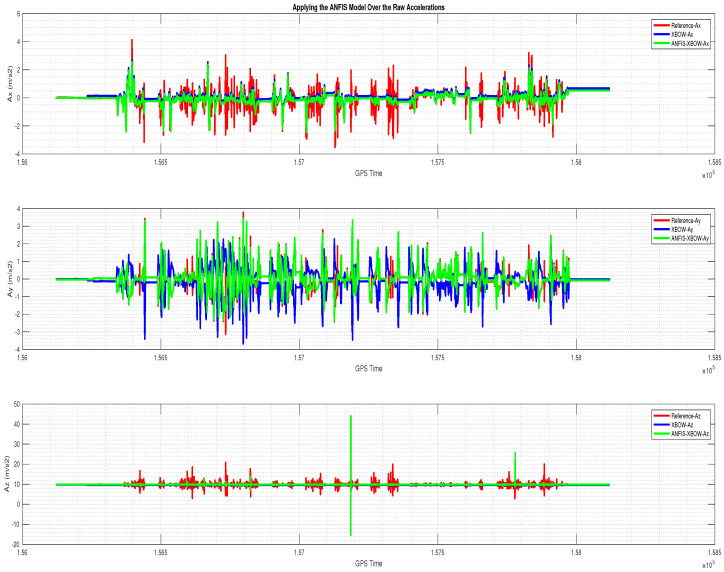
The 3D accelerometers with the ML–based–ANFIS (testing stage).

**Figure 12:**
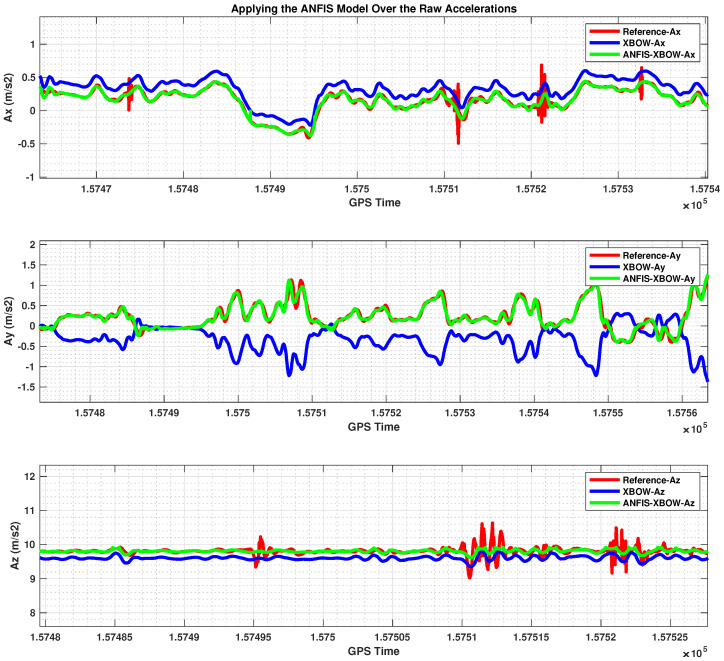
A zoomed–in part of the IMU accelerometers.

**Figure 13:**
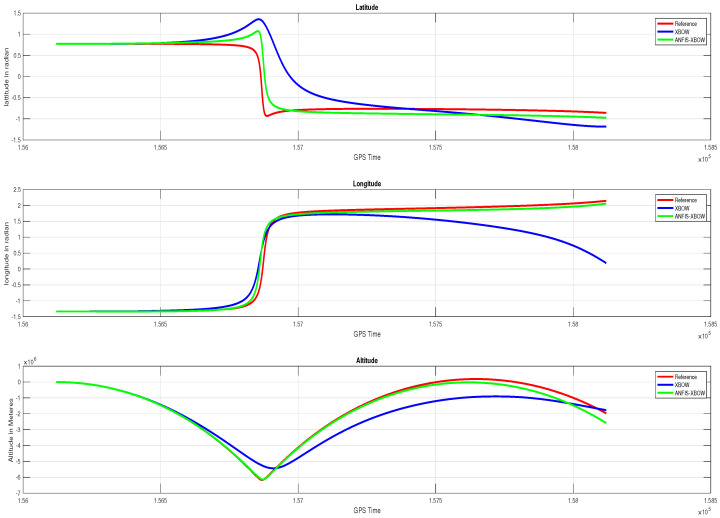
Position (Lat, Long, and Alt) components’ comparison.

**Figure 14:**
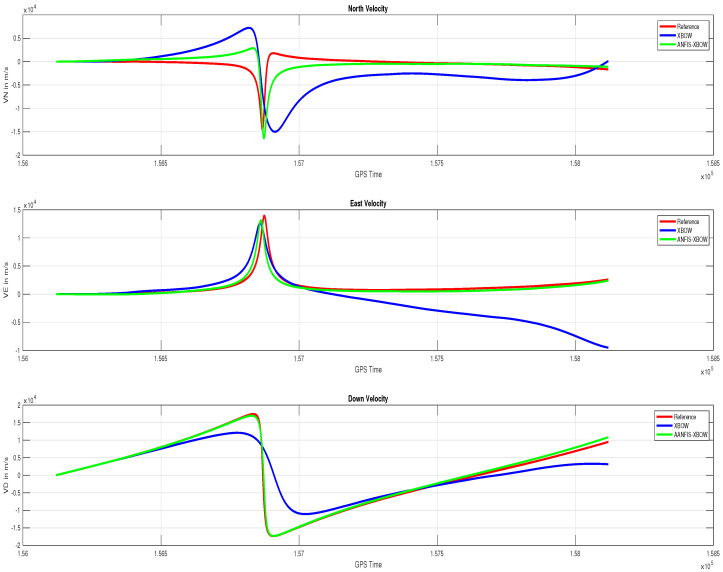
Velocity ($V_{N}$, $V_{E}$, and $V_{D}$) components’ comparison.

**Figure 15:**
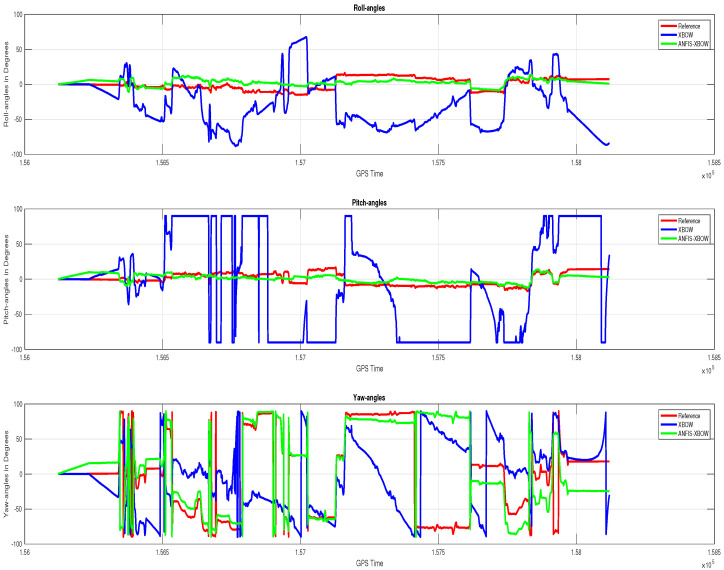
Attitude (roll, pitch, and yaw) angles’ comparison.

The application of the ML-based-ANFIS to the XBOW IMU measurements was carried out in two stages. The first stage was the training stage, in which the IMU-CPT was utilized as a learning source. This stage was applied with 50% of the data to generate the ML-based-ANFIS model. Three gyroscopes and three accelerometers were trained in this stage to produce a suitable model. The ML-based-ANFIS utilized six membership functions with an adaptive step size. In the results shown in Figure 7, Figure 8, Figure 9, Figure 10, Figure 11, Figure 12, Figure 13, Figure 14 and Figure 15, the reference is designated by red color, the XBOW-IMU in blue, and the proposed ML-based-ANFIS in green for both raw measurements and INS solution comparisons.

The 3D gyroscope and accelerometer measurements in the training stage are shown in Figure 7 and Figure 8, respectively.

The generated ML-based-ANFIS model was then applied to the remaining XBOW-IMU measurements. This step was the testing stage where the generated model was applied and its performance measured.

Figure 9 shows a comparison between the raw XBOW-IMU gyroscope measurements in three directions (x, y, and z) and those with the applied ML model compared to the reference angular rates from the IMU-CPT. A zoomed-in view for a portion of the testing data is shown in Figure 10. The results in Figure 10 show that the biases and scale factor and a significant part of the associated noise were removed when applying the proposed ML technique to the low-grade IMU gyro measurements.

The accelerations’ comparison for the testing part is shown in Figure 11. Additionally, a zoomed-in view for the accelerations in this stage is shown in Figure 12. The results showed the proposed ML technique’s ability to estimate and remove the errors associated with the low-grade IMU measurements. Furthermore, not only was the noise mostly removed, but both the bias and scale factor errors were also reduced significantly. Therefore, the produced IMU measurements from the proposed ML technique provided a more robust input to the INS mechanization, which led to a more accurate navigation solution.

To validate the resulting measurements, a comparison of the raw XBOW-IMU measurements before and after applying the proposed ML technique is shown in Table 2 using the RMSE for each measurement. The results showed a significant improvement of the IMU measurements when using the ML-based-ANFIS.

The output of this process was new IMU measurements that were ready to be applied to the INS algorithm. Consequently, to verify the performance of the proposed ML-based-ANFIS, the modified and unmodified measurements were applied to the INS algorithm to produce the navigation information PVA. The output PVA was then compared to the reference PVA to check the improvement and the worthiness of using ML in training on the raw MEMS-IMU measurements by a high-end IMU.

The results showed the INS solution produced from the XBOW IMU (low-grade) and the corresponding modified measurements (ML-based-ANFIS) when using the proposed ML technique compared to the reference solution from the IMU-CPT.

The position components’ (latitude in radians, longitude in radians, and altitude in meters) comparison is shown in Figure 13. The results showed that the position components when using the proposed ML technique were closer to the reference position components compared to the ones when using the raw XBOW IMU measurements. Therefore, the utilization of ML to improve the IMU measurements leveraged the position solution of the unit.

The comparison of the velocity components in the navigation frame ($V_{N}$, $V_{E},$ and $V_{D}$) are shown in Figure 14.

The results showed that there was a significant improvement in all the velocity components when using the proposed ML technique. A comparison of the attitude components’ (roll, pitch, and yaw) angle is shown in Figure 15, illustrating the significant improvement of the attitude components’ solution.

**Figure 16:**
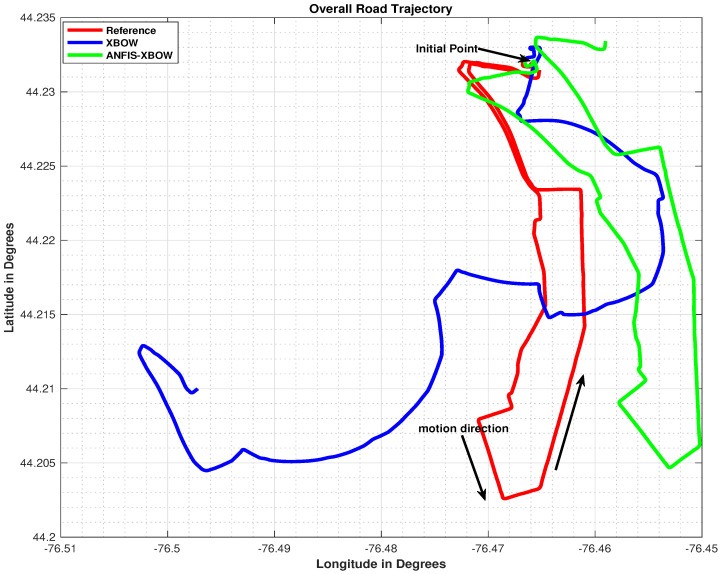
Overall trajectory comparison.

The overall trajectory comparison is shown in Figure 16. The trajectory shows the 2D position information obtained by applying the reference high-end IMU-CPT, the ow-end XBOW IMU, and the ML-based-ANFIS XBOW IMU to the INS mechanization in red, blue, and green, respectively. Furthermore, arrows show the start point and the direction of motion. The trajectory from the low-end XBOW IMU severely drifted over time compared to the one generated from the proposed ML-based-ANFIS XBOW. The result of the proposed method showed the effectiveness of using the ML-based-ANFIS technique in improving the performance of the INS navigation solution. Moreover, the proposed method trajectory followed the reference even during maneuvers with a smaller shift compared to the original XBOW one. This result came from the great enhancement of the XBOW IMU measurements after applying the proposed ML technique.

A statistical analysis of the INS solution position, velocity, and attitude components in the LLF from the testing part of the trajectory (24 min) is shown in Table 3.

A 70% overall improvement of the 2D position and 92% improvement of the 2D velocity were achieved when using the proposed ML-based-ANFIS technique. Moreover, the attitude components had a great improvement. The roll angle RMSE was reduced from 55.6 degrees to 6 degrees with an improvement of 89.2%; the pitch angle RMSE was reduced from 42.6 degrees to 6.5 degrees with an improvement of 84.7%; the yaw angle was reduced from 86.3 degrees to 79.3 degrees with an improvement of 8%. The yaw angle’s improvement percentage was less than other attitude components due to the proximity of the raw $w_{z}$ to the reference $w_{z}$, as shown in Figure 9 and Figure 10. The results showed the superiority of applying ML to leverage the low-grade IMU, which significantly enhanced the INS navigation solution compared to the traditional solution using the raw measurements of the low-grade IMU.

## 6. Conclusions and Future Work

This paper discussed the utilization of the ML-based-ANFIS to improve the raw MEMS-grade IMU measurements. The proposed ML algorithm was applied to real data collected with a low-cost IMU. The proposed ML technique was applied to 50% of the collected data and tested on the remaining data. The output of this process was then applied to a strap-down INS to produce a navigation solution PVA. The produced navigation solution achieved a 2D position improvement of 70% and a 2D velocity improvement of 92%. Furthermore, an improvement of 89.2%, 84.7%, and 8% of the attitude components of the roll, pitch, and yaw, respectively, was achieved. The work in this paper showed that using ML to boost a low-grade IMU had a significant impact on the inertial sensors’ performance. Moreover, it had a great impact by producing a more accurate and robust INS navigation solution. As a future step, this work can be combined with either another ML technique or the EKF to bridge GNSS outages in challenging GNSS environments.

## Figures and Tables

**Table 1:** Utilized IMUs’ performance characteristics.

IMUs	IMU300CC(XBOW)	IMU-CPT
	(100 HZ)	(100 Hz)
Size (cm${}^{3}$)	7.62 × 9.53 × 3.2	15.2 × 16.8 × 8.9
Weight	0.59 Kg	2.28 Kg
Max data rate	200 Hz	100 Hz
Start-up time	<1 s	<5 s
Accelerometer		
Range	±2 g	±10 g
Bias instability	±30 mg	±0.75 mg
Scale factor	<1%, 1σ	300 ppm, 1σ
Gyroscope		
Range	±100${}^{\circ }$/s	±375${}^{\circ }$/s
Bias instability	<±2.0${}^{\circ }$/s	±1.0${}^{\circ }$/h
Scale factor	<1%, 1σ	1500 ppm, 1σ

**Table 2:** IMU raw measurements’ RMSE comparison.

RMSE	$\omega_{x}$	$\omega_{y}$	$\omega_{z}$	$a_{x}$	$a_{y}$	$a_{z}$
XBOW	0.0158	0.0213	0.0064	0.1902	1.2629	0.3542
ML-XBOW	0.0084	0.0069	0.0026	0.1084	0.0757	0.2873

**Table 3:** Results’ analysis of the testing part of the trajectory (24 min).

Units		XBOW	ML-XBOW
Position RMSE (m)	North	311,899.4	222,857.2
	East	732,549.2	83,613.3
	Down	967,706.2	291,313.6
	2D Pos	796,184.3	238,026.2
	3D Pos	1,253,141.9	376,191.6
Velocity RMSE (m/s)	VN	2730.5	360.5
	VE	5879.4	338.9
	VD	2242.8	655.2
	2D Vel	6482.5	494.8
	3D Vel	6859.5	821
Attitude RMSE (Deg)	Roll	55.6	6.09
	Pitch	42.6	6.5
	Yaw	86.3	79.3
